# The optimal extent of lymph node dissection in gastroesophageal junctional cancer: retrospective case control study

**DOI:** 10.1186/s12885-019-5922-8

**Published:** 2019-07-22

**Authors:** Won Ho Han, Bang Wool Eom, Hong Man Yoon, Daniel Reim, Young-Woo Kim, Moon Soo Kim, Jong Mog Lee, Keun Won Ryu

**Affiliations:** 1 Center for Gastric Cancer, Research Institute & Hospital, National Cancer Center, 323 Ilsan-ro, Ilsandong-gu, Goyang-si, 410-769 Republic of Korea; 2 Department of Surgery, Technical University of Munich, School of Medicine, Klinikum rechts der Isar, Ismaninger Straße 22, 81675 Munich, Germany; 3 Center for Lung Cancer, Research Institute & Hospital, National Cancer Center, 323 Ilsan-ro, Ilsandong-gu, Goyang-si, 410-769 Republic of Korea

**Keywords:** Gastroesophageal junction cancer, Gastric cancer, Lymphadenectomy, Mediastinal lymph node dissection, Siewert type

## Abstract

**Background:**

Recently, the incidence of gastroesophageal junction (GEJ) cancer has been increasing in Eastern countries. Mediastinal lymph node (MLN) metastasis rates among patients with GEJ cancer are reported to be 5–25%. However, survival benefits associated with MLN dissection in GEJ cancer has been a controversial issue, especially in Eastern countries, due to its rarity and potential morbidity.

**Methods:**

We retrospectively reviewed 290 patients who underwent surgery for GEJ cancer at the National Cancer Center in Korea from June 2001 to December 2015. Clinicopathologic characteristics and surgical outcomes were compared between patients without MLN dissection (Group A) and patients with MLN dissection (Group B). Prognostic factors associated with the survival rate were identified in a multivariate analysis.

**Results:**

Twenty-nine (10%) patients underwent MLN dissection (Group B). Three of 29 patients (10.3%) showed a metastatic MLN in Group B. For abdominal LNs, the 5-year disease-free survival rate was 79.5% in Group A and 33.9% in Group B (*P* < 0.001). The multivariate analysis revealed that abdominal LN dissection, pT category, and pN category were statistically significant prognostic factors. LNs were the most common site for recurrence in both groups.

**Conclusion:**

Abdominal LN dissection and pathologic stage are the important prognostic factors for type II and III GEJ cancer rather than mediastinal lymph node dissection.

## Background

While gastroesophageal junction (GEJ) cancer has been commonly observed in Western countries, the incidence of GEJ cancer is still rare but has been increasing in Eastern countries in recent years [1, 2]. The Siewert classification system is widely used to classify GEJ cancer according to the distance from the tumor epicenter to the GE junction [3]. However, there is a controversy regarding whether GEJ cancer should be classified as gastric or esophageal cancer [4]. The American Joint Committee on Cancer/Union for International Cancer Control (AJCC/UICC) staging (7th edition, published in 2010) classifies Siewert type I and II as esophageal cancer and type III involving the GEJ as esophageal cancer [4]. However, the 8th edition (published in 2017) classifies Siewert type II as esophageal cancer, and Siewert type III was changed to gastric cancer [5].

Due to the vague anatomical location of GEJ cancer, the range of esophagogastric resection, the staging system, and the extent of lymph node dissection, including mediastinal lymph nodes (MLNs) for this disease entity have been controversial [3, 4]. MLN metastasis rates among patients with GE junction type II and III adenocarcinoma are reported to be 5–25% [6–9]. However, MLN dissection is rarely performed in Eastern countries due to the rarity of type I and its invasiveness and associated morbidity. Furthermore, whether MLN dissection has survival benefits has been a debatable issue. [6, 10–13]

In this study, we investigated the optimal extent of LN dissection in GEJ cancer via the analysis of the distribution of lymph node metastasis, prognostic factors and recurrence patterns in GEJ cancer.

## Methods

A total of 290 patients who were diagnosed with GEJ adenocarcinoma at the National Cancer Center in Korea between June 2001 and December 2015 and underwent curative resection were included. Multiple primary gastric cancer at initial diagnosis, recurrent gastric cancer after curative gastrectomy patients, and those with a history of preoperative chemotherapy were excluded.

Clinicopathologic factors and surgical outcomes of enrolled patients were retrospectively analyzed. Included in the analysis were the patient’s age, sex, preoperative BMI, co-morbidity represented by the American Society of Anesthesiologist (ASA) score, tumor size, location, extent of LN dissection, number of harvested and metastatic LNs, differentiation, Lauren’s classification, surgical procedures, stage, postoperative complications, adjuvant chemotherapy, recurrence status and location.

The study population was classified into patients without MLN dissection (Group A) and those with MLN dissection (Group B). Siewert’s classification was based on the distance from the tumor epicenter to the GEJ measured by preoperative endoscopic examination or the pathologic report obtained after surgery [14]. The dissected LN station and status of lymph node metastasis were investigated in both groups. The LN classification was determined according to Japanese gastric cancer treatment guidelines. [15] Complications were classified and graded according to the Clavien-Dindo classification [16]. The initial recurrence site was defined as the location where the first recurrence was found on postoperative CT or endoscopy. To evaluate the risk factors for the disease-free survival rate, extent of lymph node dissection, age, sex, Siewert type, tumor size, histology, proximal margin, stage, and adjuvant chemotherapy were included in the multivariate analysis.

Endoscopy and abdominopelvic CT were performed every 6 months for 5 years post-surgery, and an endoscopy was performed annually for 5 years post-surgery. Recurrence patterns were classified as locoregional, peritoneal, and hematogenous metastasis. This study was approved by the Institutional Review Board of the National Cancer Center (No.NCC2017–0224).

Clinical and pathological variables were analyzed using the χ^2^ test (or Fisher’s exact test) and Student’s t-test for normally distributed continuous data. Univariable analyses of the survival rate were conducted using the log-rank test. All variables with a univariable *P*-value*<* 0·05 were included in the multivariable analysis using a Cox proportional hazards model. Variables with a *P*-value < 0·05 were considered statistically significant. All analyses were performed using SAS® version 9.1.3 for Windows® (SAS Institute, Cary, North Carolina, USA).

## Results

### Patient demographics and surgical outcomes

Of the 290 total patients, 29 (10%) patients underwent MLN dissection (group B) (Table 1). The proportion of patients classified as Siewert type II was higher in group B (39.5% for Group A vs. 62.1% for Group B, *p* = 0.019). In Group B, the tumor size was larger (4.4 ± 2.5 for Group A vs. 5.7 ± 2.9 for Group B, *p* = 0.025), more invasive (pT category *p* = 0.035), and more commonly involved LN metastasis (pN category *p* = 0.006). While 12 patients (41.4%) underwent esophagectomy (Ivor Lewis) in group B, none of the group A patients underwent esophagectomy. The proximal margin was significantly longer in group B (1.9 ± 1.1 in Group A vs. 4.6 ± 4.9 in Group B, *p* < 0.001). Abdominal D2 or additional LN dissection was performed more frequently in Group A patients. The number of patients who underwent adjuvant chemotherapy was also higher for group B.

**Table 1:** Demographics of gastroesophageal junction cancer patients

	Patients without MLND Group A (*N* = 261)	Patients with MLND Group B (*N* = 29)	Value
Age	60.6 ± 12.1	61.4 ± 11.0	0.751
Sex			0.641
Male	200 (76.6%)	24 (82.8%)	
Female	61 (23.4%)	5 (17.2%)	
BMI*	23.6 ± 3.5	23.0 ± 3.5	0.492
ASA score**			0.809
0	82 (31.4%)	10 (34.4%)	
1	162 (62.0%)	17 (58.6%)	
2 or more	17 (6.5%)	2 (6.8%)	
Siewert Type			0.019
Type II	103 (39.5%)	18 (62.1%)	
Type III	158 (60.5%)	11 (37.9%)	
Tumor size	4.4 ± 2.5	5.7 ± 2.9	0.025
Surgical procedure			< 0.001
Total gastrectomy	238 (91.2%)	16 (55.2%)	
Proximal gastrectomy	23 (8.8%)	1 (3.4%)	
Esophagectomy (Ivor Lewis)	0 (0%)	12 (41.4%)	
Splenectomy			1.000
Yes	17 (6.5%)	1 (3.4%)	
No	244 (93.5%)	28 (96.6%)	
Histopathological type			0.064
Differentiated	103 (39.8%)	16 (56.2%)	
Undifferentiated	142 (54.8%)	13 (44.8%)	
Others	14 (5.4%)	0 (0%)	
Lauren classification			0.006
Intestinal	144 (55.2%)	12 (41.4%)	
Diffuse	79 (30.3%)	8 (27.6%)	
Mixed	24 (9.2%)	2 (6.9%)	
Unknown	14 (5.4%)	7 (24.1%)	
Proximal margin	1.9 ± 1.1	4.6 ± 4.9	< 0.001
Extent of Abdominal			< 0.001
LN dissection			
D1+	36 (13.8%)	15 (51.7%)	
D2 or more	225 (86.2%)	14 (48.3%)	
Harvested LNs	42.4 ± 16.7	43.1 ± 14.6	0.827
Metastatic LNs	3.0 ± 6.4	5.8 ± 6.7	0.043
T category			0.035
pT1	107 (41.0%)	6 (20.7%)	
pT2	45 (17.2%)	5 (17.2%)	
pT3	70 (26.8%)	12 (41.4%)	
pT4	39 (14.9%)	6 (20.7%)	
pN category			0.006
pN0	158 (60.5%)	6 (20.7%)	
pN1	34 (13.0%)	11 (37.9%)	
pN2	26 (10.0%)	5 (17.2%)	
pN3	43 (16.5%)	7 (24.1%)	
Cytology			0.027
negative	260 (99.6%)	27 (93.1%)	
Positive	1 (0.4%)	2 (6.9%)	
Stage***			< 0.001
Stage I	147 (56.3%)	6 (20.7%)	
Stage II	54 (20.7%)	12 (41.4%)	
Stage III	57 (21.8%)	8 (27.6%)	
Stage IV	3 (1.1%)	3 (10.3%)	
Adjuvant ChemoTx	87 (33.3%)	20 (68.9%)	< 0.001

* *MLND* mediastinal lymph node dissection

*BMI body mass index (kg/m^2^)

**ASA American Society of Anesthesiologists

*** AJCC 7th edition: Esophagus and Esophagogastric Junction

### LN dissection and metastasis

Distribution of metastatic lymph nodes among the dissected lymph nodes in each LN station was compared between the groups (Table 2). Of the 261 patients in group A, the lymph node stations were not classified in 54 patients. Three patients in group B (10.3%) showed metastatic MLNs. All these patients were Siewert type II patients, and one patient had metastasis of the lower and upper mediastinum simultaneously.

**Table 2:** Comparison of LN metastasis based on lymph node station

Group A (*N* = 207^a^)				Group B (*N* = 29)		
LN station	No. of Patients with Metastatic LNs	No. of Patients with LN dissection	Percent (%)	No. of Patients with Metastatic LNs	No. of Patients with LN dissection	Percent (%)
Upper mediastinum	0	0	0	1	12	8.3
Middle mediastinum	0	0	0	0	11	0
Lower mediastinum	0	0	0	3	29	10.3
1	38	207	18.3	8	14	57.1
2	31	196	15.8	5	11	45.4
3	31	207	14.9	9	14	64.2
4d	4	190	2.1	1	10	10.0
4sa	4	191	2.1	2	9	22.2
4sb	5	203	2.4	1	11	9.0
5	3	196	1.5	0	9	0
6	2	197	1.0	0	12	0
7	19	191	9.9	4	14	28.5
8	9	119	7.5	1	8	12.5
9	15	189	7.9	4	11	36.3
10	5	95	5.2	0	4	0
11p	11	171	6.4	1	7	14.2
11d	4	128	3.1	0	5	0
12a	4	146	2.7	0	10	0
Para aortic LN	3	9	33.3	0	2	0

^a^ Of the 261 patients in group A, lymph node station were not classified in 54 patients

In group A, the rate of abdominal LN metastasis of LN #1 (17.43%), #2 (15.81%), #3 (14.97%) and #7 (9.94%) was high, whereas group B had a higher rate of LN metastasis in all areas except the distal stomach (LN #5 and #6), splenic region (LN #10 and #11d) and LN #12a.

### Postoperative complications

Surgical complication rates were 37.9% in group B and 30.3% in group A (Table 3). Severe complications (>Clavien-Dindo grade II) were detected in 4 (13.8%) and 31 (11.9%) cases (*p* = 0.397). Respiratory complications were significantly higher in group B (24.1%) compared to those in group A (7.3%) (*p* = 0.003). Postoperative mortality was 3.4 and 1.1%, respectively (*p* = 0.345).

**Table 3:** Postoperative Complications

	Group A (*N* = 261)	Group B (*N* = 29)	Value
All complication	79 (30.3%)	11 (37.9%)	0.397
Severe complication (above CD grade III)^a^	31 (11.9%)	4 (13.8%)	0.764
Anastomosis related complication (leakage, stricture)	23 (8.8%)	5 (17.2%)	0.145
Respiratory realated			
Complication (pneumonia, pleural effusion, pneumothorax	19 (7.3%)	7 (24.1%)	0.003
Postoperative mortality	3 (1.1%)	1 (3.4%)	0.345

^a^*CD* clavien dindo classification

### Multivariable analysis of prognostic factors

**Fig. 1:**
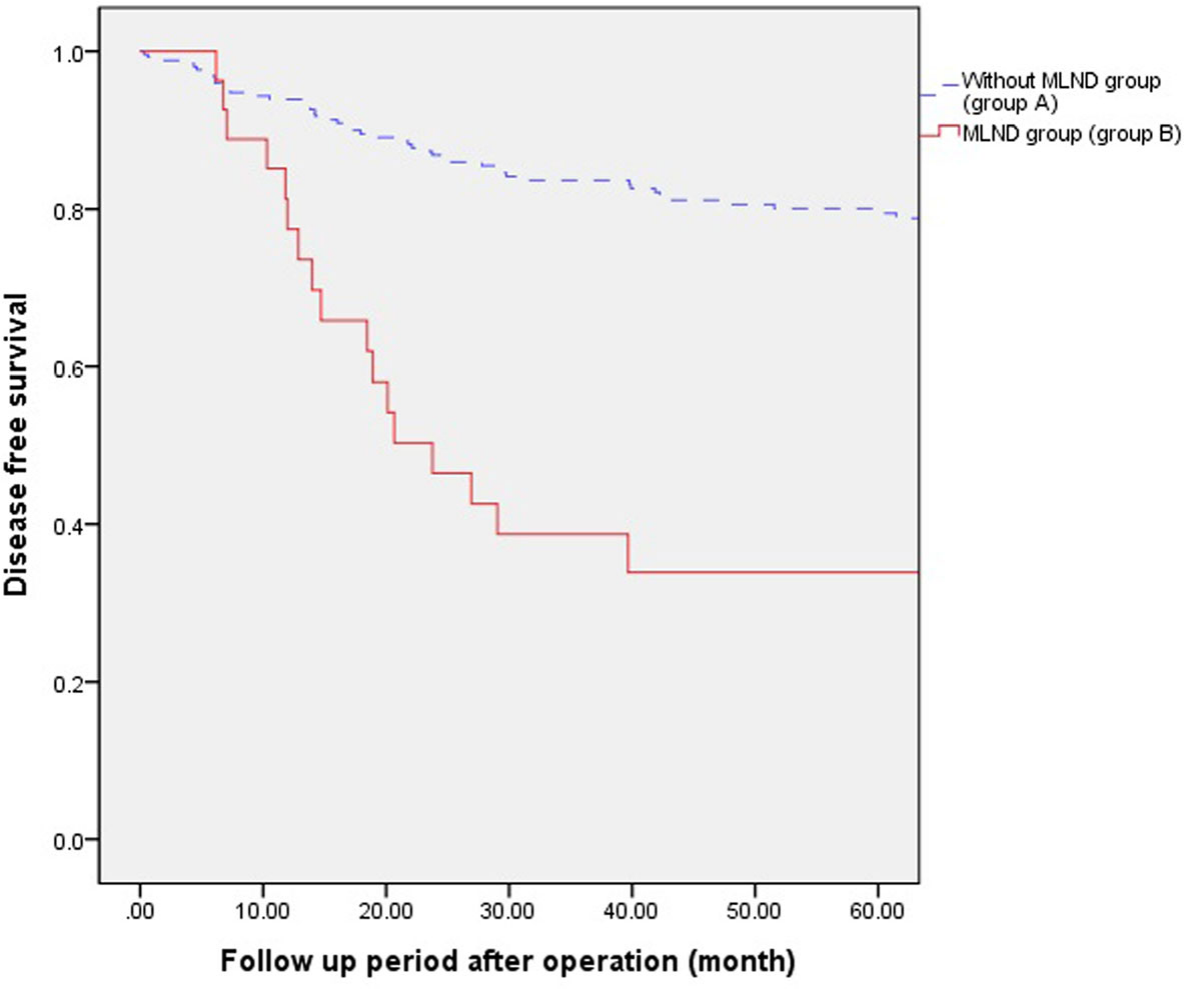
Five years Disease free survival and overall between group A and B. The five-year disease-free survival rate was 79.5% in group A and 33.9% in group B (*P* < 0.001)

**Fig. 2:**
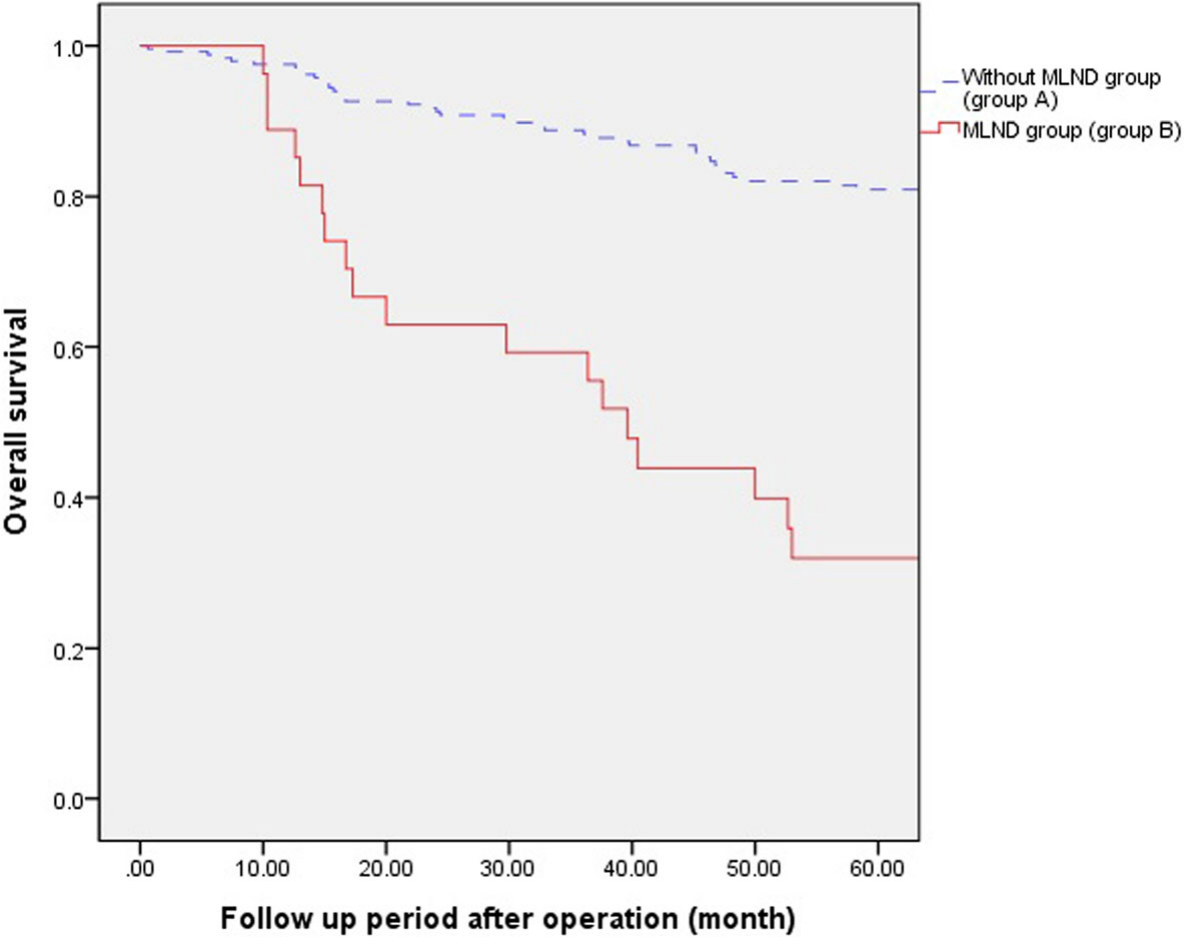
Five years Disease free survival and overall between group A and B. The five-year overall survival rate was 80.9% in group A and 31.9% in group B (*P* < 0.001)

**Fig. 3:**
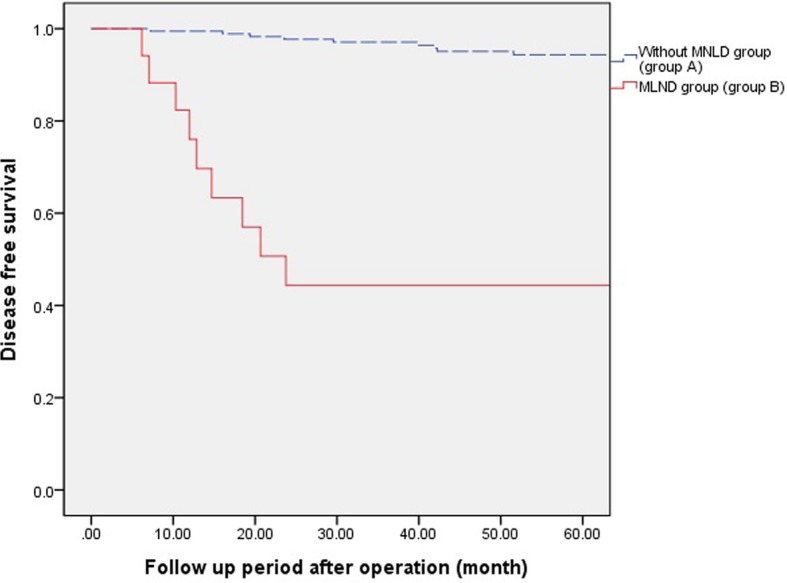
Five years Disease free survival according to pathologic stage. The five-year disease-free survival rate was 94.3% in group A and 42.5% in group B (*P* < 0.001)

**Fig. 4:**
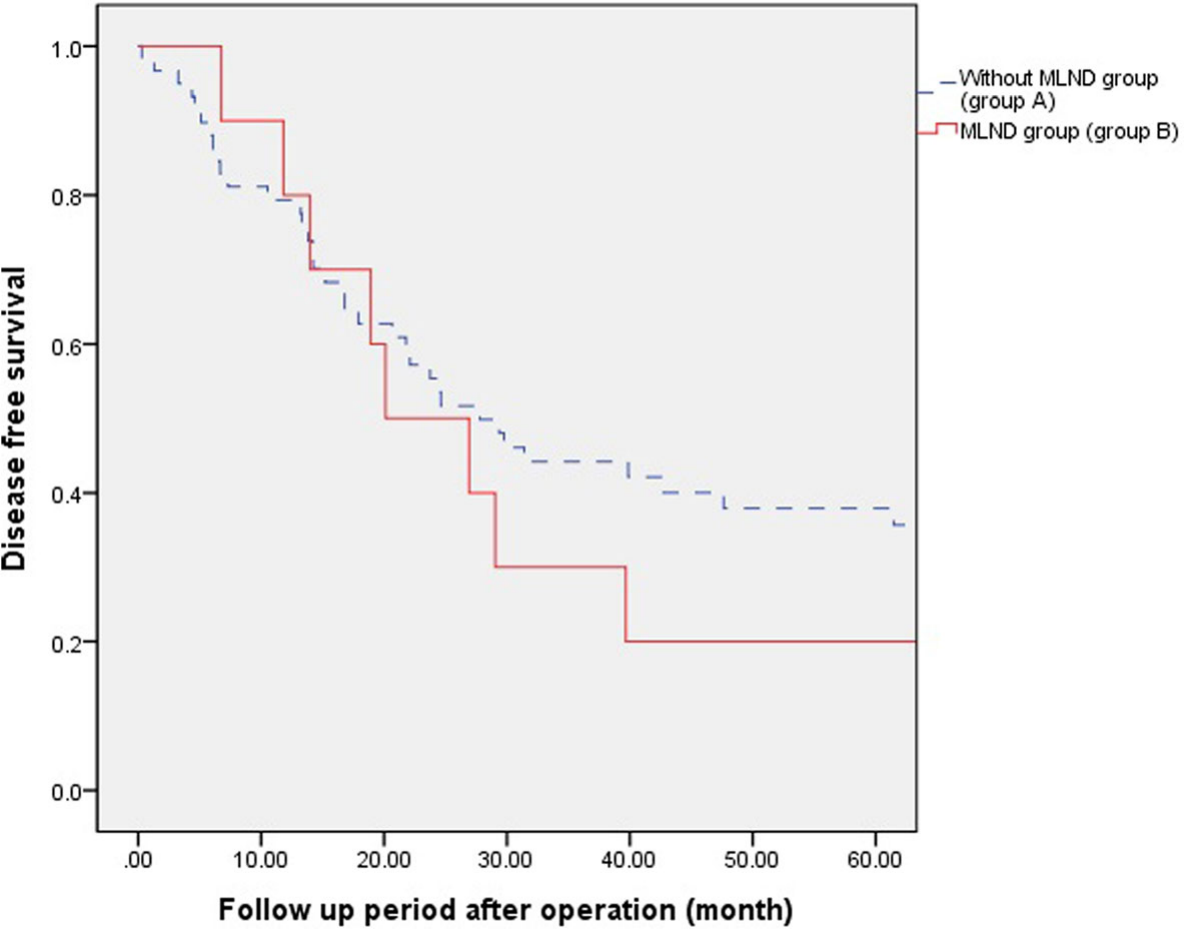
Five years Disease free survival according to pathologic stage. However there was no difference in survival between the two groups in pStage III,IV (37% vs 20% *p* = 0.433)

The five-year disease-free survival rate was 79.5% in group A and 33.9% in group B (*P* < 0.001) (Fig. 1). The five-year overall survival rate was 80.9% in group A and 31.9% in group B (*P* < 0.001) (Fig. 2). Two hundred eighty-seven patients were included in the survival analyses, excepting 3 patients who were included in the analysis for postoperative mortality within 30 days. The five-year disease-free survival rate was 94.3% in group A and 42.5% in group B (*P* < 0.001) (Fig. 3). However there was no difference in survival between the two groups in pStage III,IV (37% vs 20% *p* = 0.433) (Fig. 4).

In the univariate analysis, MLN dissection, D1 + dissection of abdominal LNs, longer tumor size, higher pT category, pN category and pM category, undifferentiated histology, and treatment with chemotherapy were associated with statistically worse survival (Table 4). A Cox proportional hazards model indicated that the extent of abdominal LN dissection was an independent prognostic factor (HR = 3.174, CI95% 1.302–7.738 *p* = 0.011) along with pT category (HR = 2.807, CI95% 1.309–6.017 *p* = 0.008) and pN category (HR = 3.815, CI95% 1.722–8.455 *p* < 0.001).

**Table 4:** Multivariable analysis of prognostic factor (Disease free survival)

		Univariable		Multivariable	
	Number of patients	Disease free survival rates (%)	*P* value (Log rank test)	Hazard ratio (95% CI)	*P* value
Age			0.383		
< 60	165	77.0			
≥ 60	122	74.6			
Sex					
Male	221	75.6	0.815		
Female	66	77.3			
Mediastinal LN dissection			< 0.001		0.328
No	259	79.5		1	
Yes	28	33.9		1.473 (0.678–3.199)	
Abdominal LN dissection			0.043		0.011
D2 or more	238	81.4		1	
D1+	49	65.3		3.174 (1.302–7.738)	
Siewert Type			0.870		
Type II	120	79.2			
Type III	167	73.7			
Tumor size			< 0.001		0.731
< 4 cm	151	86.8		1	
≥ 4 cm	136	64.0		1.113 (0.487–1.656)	
pT category			< 0.001		0.008
pT1, T2	160	90.0		1	
pT3, T4	217	58.3		2.807 (1.309–6.017)	
pN category			< 0.001		< 0.001
pN0	163	93.9		1	
pN+	124	52.4		3.815 (1.722–8.455)	
pM category^a^			< 0.001		0.070
pM0	282	77.3		1	
pM1	5	0.0		2.754 (0.920–8.241)	
Histology			0.025		0.109
Differentiated	117	82.1		1	
Undifferentiated	155	74.2		1.469 (0.918–2.350)	
Proximal margin			0.445		
≥ 2 cm	140	76.4			
< 2 cm	147	75.5			
Chemotherapy			< 0.001		0.096
No	179	90.5		1	
Yes	108	51.9		1.751 (0.905–3.388)	

^a^pM category: 3cases were diagnosed with washing cytology positive and 3cases were diagnosed with paraaortic LN metastasis in final pathology

### Recurrence pattern

The recurrence pattern was classified according to the site of initial recurrence (Table 5). A total of 40 patients (33.3%) in group A and 15 patients (51.7%) in group B revealed recurrences during the follow-up period. Multiple recurrences detected simultaneously were also included. LN recurrence (50%) was the most common type of recurrence in group B. LN recurrence and hematogenous metastasis occurred at the same rate (34.7%) in group A. When comparing patterns of LN recurrence, the MLN recurrence was more common in group B (50%, 4/8), whereas the paraaortic LN recurrence rate was more common (81%, 13/16) in group A.

**Table 5:** Recurrent pattern

Recurrence site	Group A (*N* = 40)^a^	Group B (*N* = 15)^b^
Locoregional	18 (39.1%)	10 (62.5%)
LN recurrence	16	8
Para aortic	13	3
Mediastinal	2	4
Perigastric	1	1
Anastomosis site	2	2
Peritoneum	12 (26.0%)	2 (12.5%)
Hematogenous	16 (34.7%)	4 (25.0%)
Liver	5	1
Lung	2	2
Bone	3	1
Colon	2	0
Kidney	1	0
Ovary	2	0
Brain	1	0

^a^In patients without Mediastinal LN dissection group (group A), recurrence was found concurrently in 6 cases. In 3 cases, paraaortic metastasis and hematogeouns metastasis were present. In 3 cases, peritomeum and hematogeous metastasis were found

^b^ In patients with Mediastinal LN dissection group (group B), there was a case in which paraaortic metastasis and bone metastasis were found concurrently

Among patients who received adjuvant chemotherapy, recurrence was significantly increased from 42.9% (12 cases) to 57.1% (16 cases) when the delay of adjuvant chemotherapy was more than 8 weeks. (*p* = 0.021).

## Discussion

In this study, the pathologic stage (pT category, pN category) and extent of abdominal LN dissection were significant prognostic factors rather than MLN dissection and the Siewert classification type in GEJ cancer. Even though patients with MLN dissection had more advanced disease and a poor prognosis, the analysis of the recurrence pattern showed that MLN dissection did not reduce MLN recurrence. In addition, the respiratory complications increased after MLN dissection. The prognostic significance of MLN dissection in GEJ cancer was not conclusive in this study.

In this study, none of the patients were diagnosed with Siewert type I adenocarcinoma. Unlike studies conducted in Western countries [3], studies in Korea and Japan reported the rates of Siewert type I cancer to be very low or close to zero in comparison with the rates of Siewert type II and III cancers [17, 18]. Consequently, concern was focused on the characteristics and treatment of Siewert type II GEJ cancer in Eastern Asian countries.

MLN metastasis rates in GE junction Type II and III adenocarcinomas are reported to be 5–25% [6–9], and postoperative MLN recurrence rates are reported to be 0–11% [6, 10, 11, 19]. In this study, none of the type III patients showed recurrence in the mediastinum. Considering that MLN dissection can increase respiratory complications in this study, MLN dissection seems to be unnecessary for type III GEJ cancer in Eastern Asian patients.

There has been controversy as to whether GEJ cancer should be classified and treated as esophageal cancer or gastric cancer [18]. The results of recent studies suggest that type II GEJ cancer should be classified as esophageal cancer including MLN dissection [14, 20]. However, in this study, MLN recurrence rates were higher in patients who underwent MLN dissection, even though more advanced-staged patients had been selected for MLN dissection. This suggests that MLN dissection might not be effective in preventing MLN recurrence in the present study. Similar results of a higher recurrence rate were found in a previous study in which patients underwent MLN dissection [11]. For this reason, further studies are needed to determine the necessity of MLN dissection in GEJ cancer.

Recent studies have reported satisfactory prognoses for early stage GEJ cancer following total gastrectomy and abdominal LN dissection, and some of these studies have reported no mediastinal recurrence after surgery [10, 19]. This suggests that sufficient abdominal LN dissection is more important than MLN dissection in GEJ cancer. However, there have been few studies comparing the prognosis according to the extent of abdominal LN dissection. The necessity of D2 dissection in GEJ adenocarcinoma should be considered based on the results of this study.

Similar to the results of previous studies [9, 16], LN metastasis rates were high for LN stations #1, 2, 3, and 7, and LN metastasis rates were low for distal stomach LNs #5 and #6 in patients with GEJ cancer (0–3.5%). The rate of LN metastasis at the suprapancreatic area (#8a, #9 and #11p) was found to be 12.5–36.3% for the MLN dissection group (group B) indicating that abdominal LN dissection is more important for advanced GEJ cancer. LN#2 is known to be important for LN dissection of GEJ cancer as it follows the left inferior phrenic artery and drains into the paraaortic LNs. Approximately 70% of patients who have paraaortic LN recurrence have been reported to show metastasis at LN#2 in the initial operation [21]. In this study, of the 15 patients who had paraaortic LN recurrence, 9 patients (60%) showed LN #2 metastasis in the initial operation.

In multivariate analysis, the pT category and pN category were independent prognostic factors rather than MLND or the Siewert classification type. In this study, no significant survival difference was found between Siewert types. A study compared survival rates and reported that tumor location was associated with cancer prognosis [22], while others reported that Siewert type was not associated with cancer prognosis and that baseline stage had a stronger influence on cancer prognosis [6].

Postoperative complications were slightly higher in mediastinal LN dissection group (group B). postoperative complications may lead to delay or omission of adjuvant chemotherapy. Recent studies suggest that delay or omission of adjuvant chemotherapy may have an impact survival in GEJ cancer [23, 24]. Although adjuvant chemotherapy showed less prognostic relevance, among patients who received adjuvant chemotherapy, recurrence was significantly increased when the delay of adjuvant chemotherapy was more than 8 weeks in the present study.

The present study has several limitations. First, it was conducted retrospectively at a single institution and thus did not include a high enough number of patients who underwent MLN dissection. Selection bias may be present as retrospective studies, and the difference in clinicopathologic characteristics between the two groups could have the possibility of affecting the outcome. Therefore, relatively few patients had MLN recurrence in this study; thus, statistical results should be interpreted with caution. Moreover, the results are not comparable to Western series because the multimodal treatment concepts, such as neoadjuvant chemotherapy or chemoradiation, are not applied in Eastern Asian patients. Furthermore, biologic and ethnic differences were not considered in this analysis.

## Conclusion

Abdominal LN dissection and the pathologic stage are the more important prognostic factors in type II and III GEJ cancer rather than MNLD. MLN dissection itself did not show prognostic significance. Optimal lymphadenectomy for the abdomen and mediastinum should be determined in future studies.

## Data Availability

The datasets and/or analysed during the current study are not publicly available but are available from the corresponding author on reasonable request.

## Abbreviations

GEJ: gastroesophageal junction
MLN: Mediastinal lymph node
